# Supplementary material for: Lipid-droplet associated mitochondria promote fatty-acid oxidation through a distinct bioenergetic pattern in male Wistar rats
Source: Nat Commun. 2023 Feb 11;14:766. doi: 10.1038/s41467-023-36432-0 (PMC9918515; doi:10.1038/s41467-023-36432-0)

Supplementary Figure 1

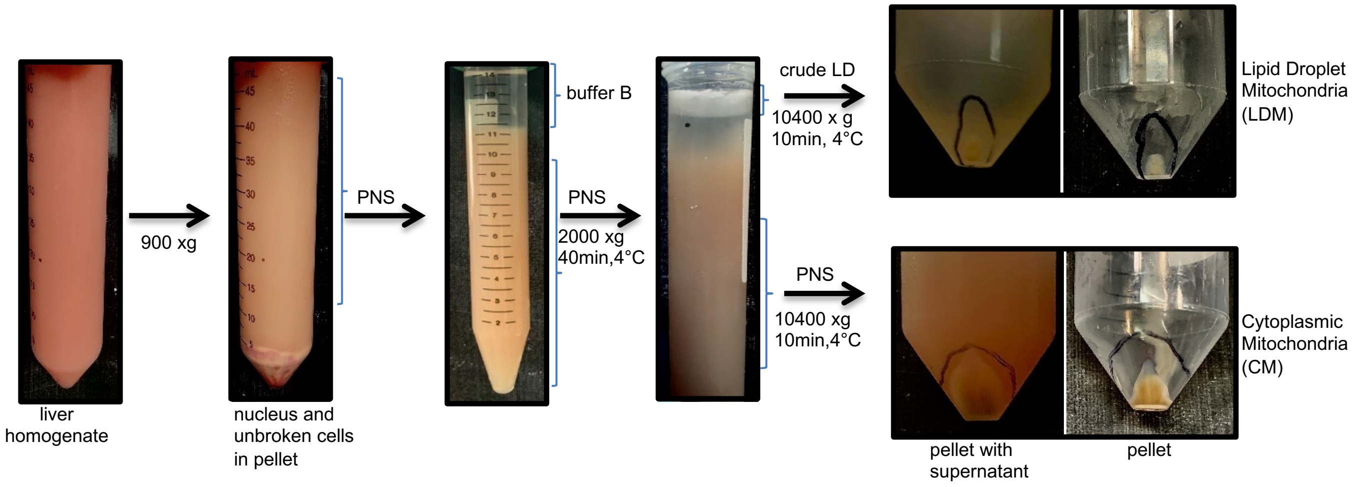

Figure S1: Pictorial illustration of the method to isolate LDM and CM from rat liver. Shown are the pictures taken during the isolation of LDM and CM from rat liver as described in the Methods section.

Supplementary Figure 2

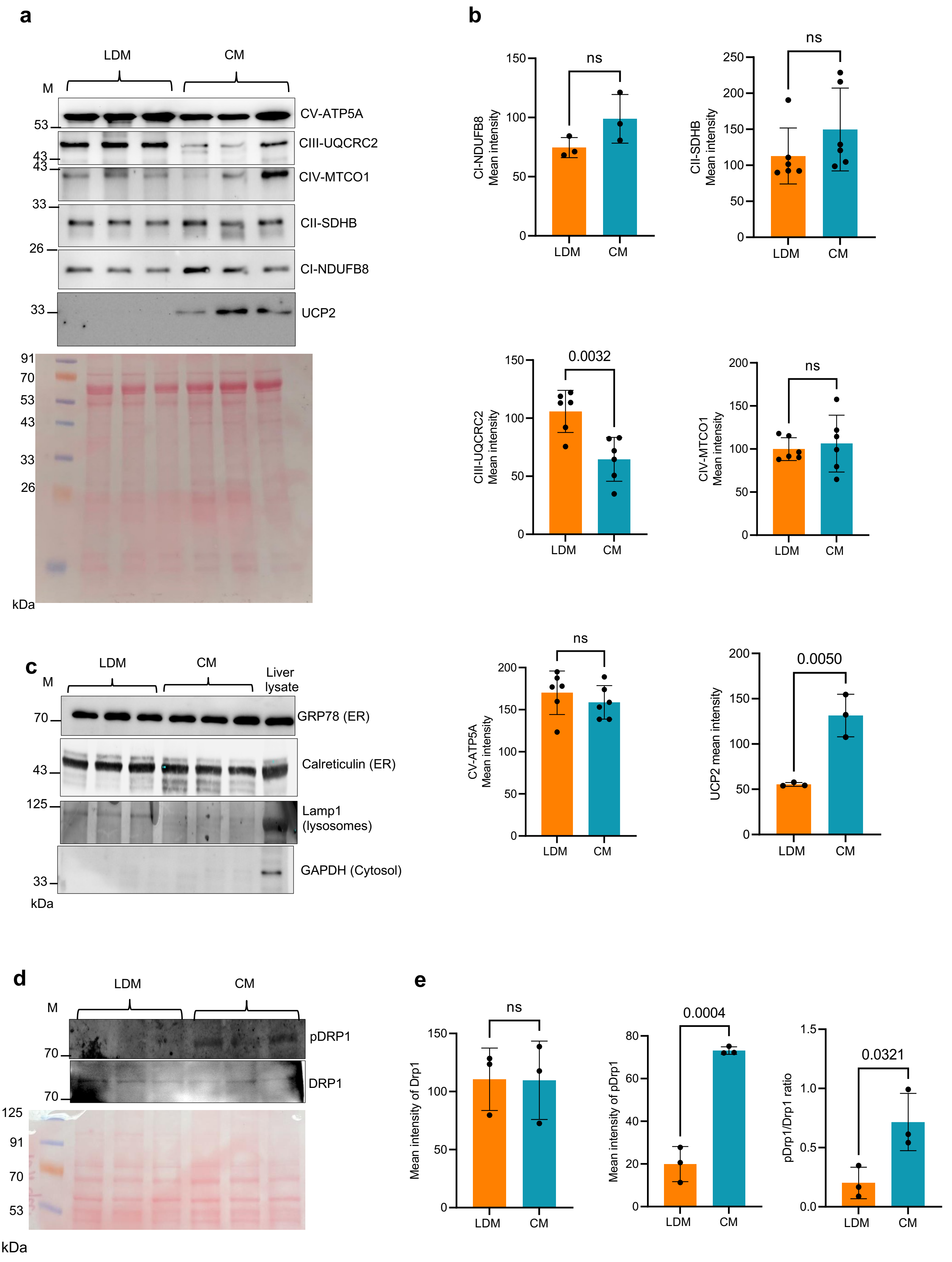

Figure S2

a) A representative Western blot probed with antibodies of OXPHOS subunits I-V (n=6) and UCP2 (n=3). The lower panel is a Ponceau S stain of the western blot.

b) Densitometric analysis of the western blot is shown in Figure S1a for isolated LDM and CM samples from the liver. Data are presented as mean values ± SD. Significance was calculated by two tailed un-paired Student t test.

c) Western blot (n=3) probed with antibodies of endoplasmic reticulum (ER) proteins GRP78 and Calreticulin, lysosomal protein Lamp1, and cytosolic marker GAPDH for isolated LDM and CM samples from the liver.

d) LDM is marked by low levels of pDRP1. Shown here is a representative Western blot (n=3) that was probed with antibodies against phosphorylated DRP1 (pDRP1) and DRP1. The lower panel is a Ponceau S stain of the western blot.

e) Quantification of Data presented in (d) panel. Data are presented as mean values ± SD. Significance was calculated by two tailed un-paired Student t test.

Supplementary Figure 3

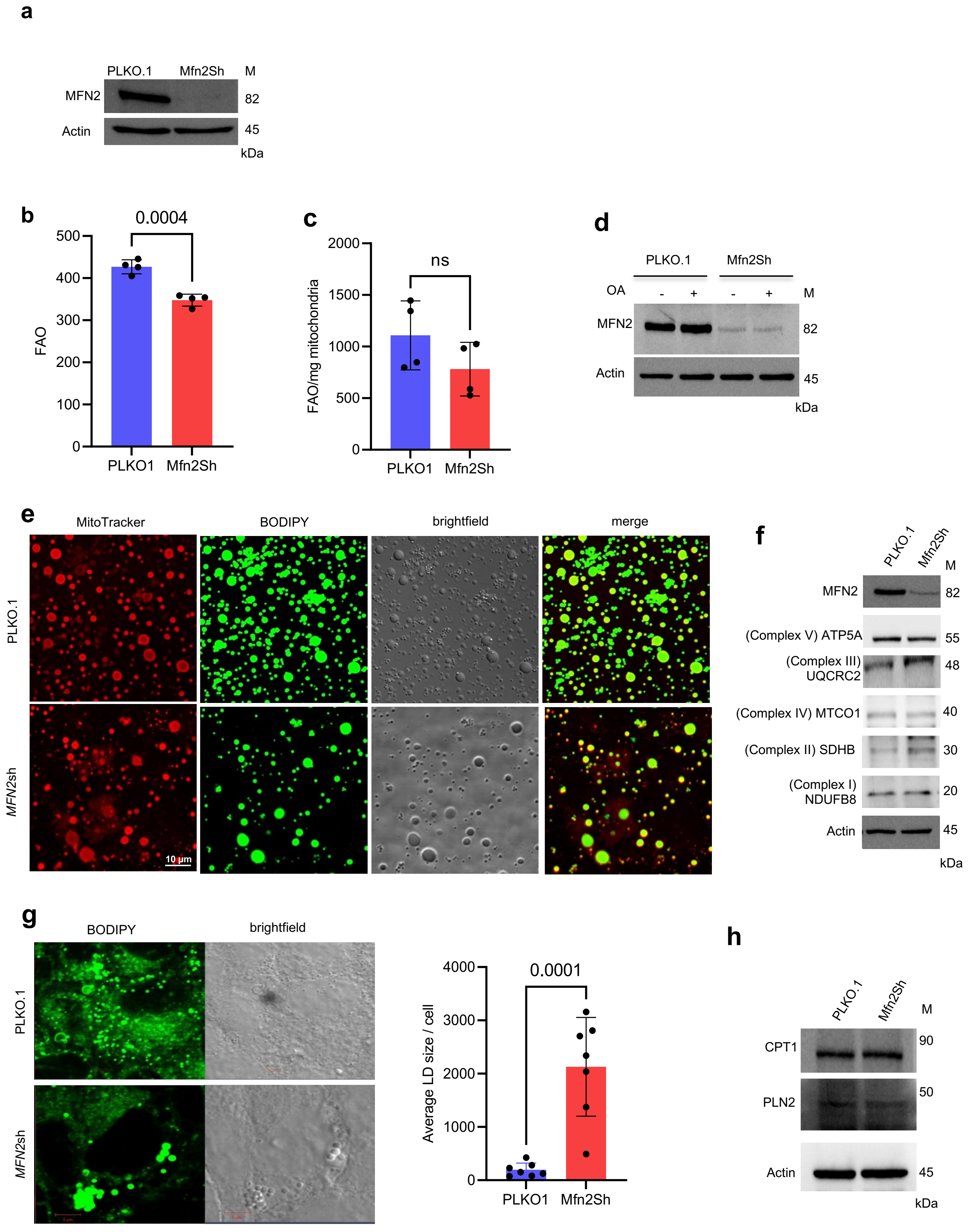

Figure S3: MFN2 increases FAO in HepG2 cells

a) Silencing of MFN2. HepG2 cells were transfected with shRNA to silence MFN2. A representative western blot probed with Mfn2 and actin (loading control) antibodies is shown of HepG2 cells transfected with the control vector (PLKO.1) or with *MFN2 shRNA* (*MFN2sh*). *MFN2* silencing was confirmed in more than three independent experiments.

b) Silencing of Mfn2 decreases FAO in HepG2 cells. FAO was monitored in HepG2 control (PLKO.1), and MFN2 silenced (*MFN2sh*) cells by carrying out the Oxygen consumption assay in the presence of 150µM Oleic acid. (n=4) Data are presented as mean values ± SD. Significance was calculated by two tailed un-paired Student t test..

c) Silencing of *MFN2* deceases FAO in mitochondria isolated from HepG2 cells. Same as in (b), except FAO was determined in whole mitochondria instead of cells. (n=4) Data are presented as mean values ± SD. Significance was calculated by two tailed un-paired Student t test.

d) Oleic acid treatment does not affect MFN2 protein expression. Same as in (a), except that the cells were treated with 0.5mM Oleic acid for 48 hrs prior to harvesting and the blot probed with antibodies against Mfn2 and actin as a loading control. Three independent experiments were performed and a representative blot is shown.

e) Preservation of LD-mitochondria contacts. Confocal images of crude LDs isolated from OA treated (0.5mM, 48hrs) HepG2 vector control cells (PLKO.1) and HepG2 cells transfected with *MFN2 shRNA* (*MFN2 sh*) and stained with MitoTracker for tracking mitochondria or with BODIPY to mark LDs (scale bar, 10 µm). Crude LD observed in minimum three independent experiments and confocal imaging was performed with minimum three technical replicates to see mitochondrial associated LD.

f) Silencing of *MFN2* does not affect the expression of OXPHOS subunits. A representative Western blot of total cell lysates from HepG2 vector control (PLKO.1) and HepG2 *MFN2 shRNA* (*MFN2 shRNA*) transfected cells probed with antibodies against MFN2, OXPHOS subunits (Complexes 1, II, IV and V) and actin as a loading control. MFN2 levels and OXPHOS levels were confirmed in three independent experiments.

g) Confocal images of HepG2 PLKO.1 and *MFN2sh* cells stained with BODIPY to mark the LDs and quantification (n=3) (scale bar, 5 µm). . Data are presented as mean values ± SD. Significance was calculated by two tailed un-paired Student t test.

h) A representative Western blot of total cell lysates from HepG2 cells probed with antibodies against CPT1, Perilipin 2 (PLN2), and actin as a loading control. Three independent experiments were performed and a representative blot is shown.

Supplementary Figure 4

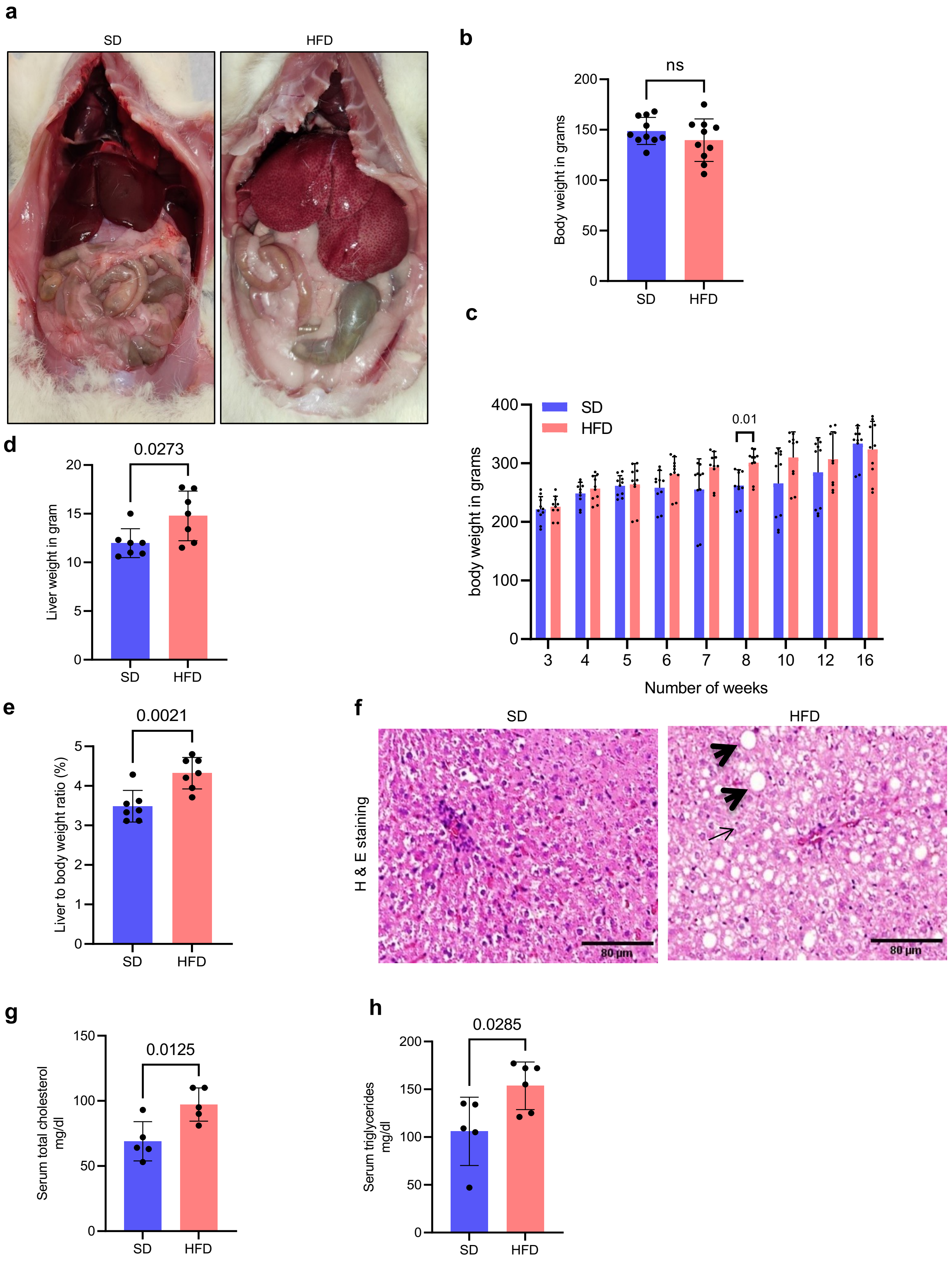

Figure S4: Histopathology of HFD and SD-fed male Wistar rats

a) Representative liver images of Wistar rats fed with SD and HFD for 16 weeks.
b) Weight match animals before the start of HFD (n=10). Data are presented as mean values ± SD. Significance was calculated by two tailed un-paired Student t test.
c) Body weight of HFD-fed animals (n=10) Data are presented as mean values ± SD. Significance was calculated by two tailed un-paired Student t test.
d-e) Liver weight and liver-to-body weight ratio in HFD-fed animals (n=7) Data are presented as mean values ± SD. Significance was calculated by two tailed un-paired Student t test.
f) Representative hematoxylin & eosin (H&E) staining images of liver sections (scale bar, 80 μm). H&E images illustrate lipid droplet accumulation, massive macro (thick arrow), and micro steatosis (thin arrow) in HFD conditions.
g-h) Total serum cholesterol and serum triglyceride levels (n=5). Data are presented as mean values ± SD. Significance was calculated by two tailed un-paired Student t test.

Supplementary information:

Standard Diet (SD) composition (Percent /weight)
1. Corn Sugar- 39.75
2. Casein Lactic- 20.00
3. Granular Sugar- 10.00
4. Dextrin- 13.20
5. Solka Floc-40- 5.00
6. AIN-93 Mineral Mix- 3.50
7. AIN-93 Vitamin Mix- 1.00
8. L-Cystine- 0.30
9. Choline Bitartrate- 0.25
10. Soy Oil- 7.00

HFD Composition (gram/kilogram)
1. Casein- 195 g/kg
2. D/L Methionine - 3 g/kg
3. Sucrose - 405.36 g/kg
4. Maltodextrin - 75 g/kg
5. Lard- 183.24 g/kg
6. Cholesterol- 12.5 g/kg
7. Soybean oil- 32.3g/kg
8. Mineral mix- 35g/kg
9. Calcium carbonate - 4 g/kg
10. Vitamin mix- 10g/kg
11. L-cysteine - 3.9g/kg
12. Choline bicarbonate - 2.6 g/kg
13. Dicalcium phosphate - 16.8 g/kg
14. Potassium citrate- 21.3 g/kg

Uncropped blot images of supplementary Figure 2

a

Cropped image kept in manuscript

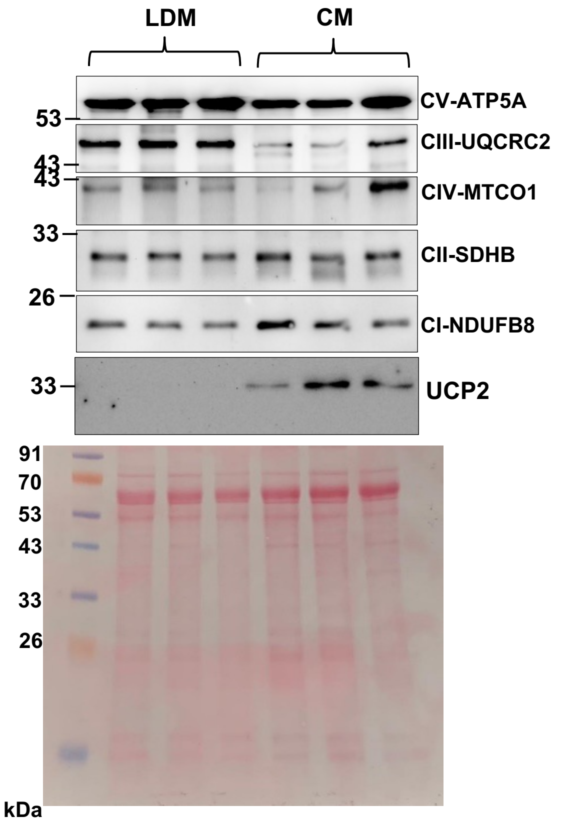

Uncropped image

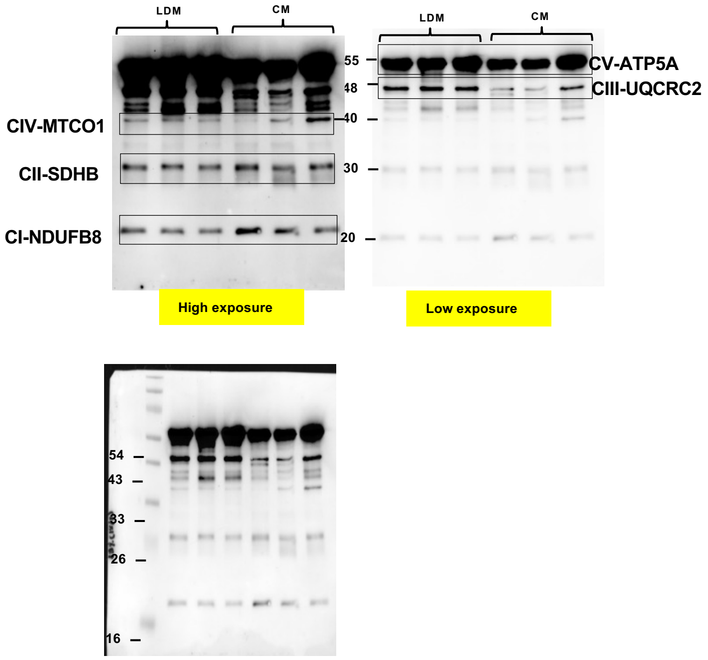

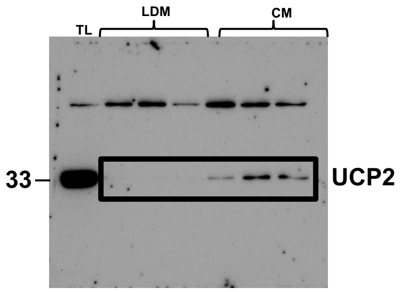

TL: tissue lysate (liver)

Uncropped blot images of supplementary Figure 2

Cropped image kept in manuscript

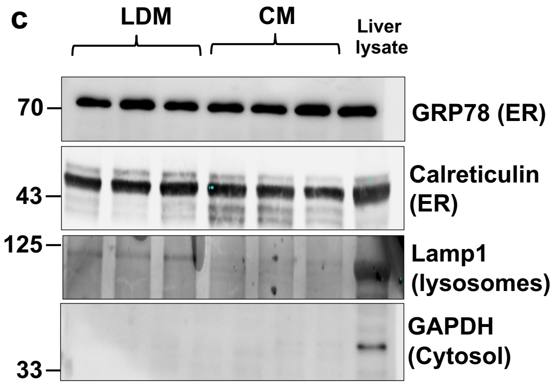

Uncropped image

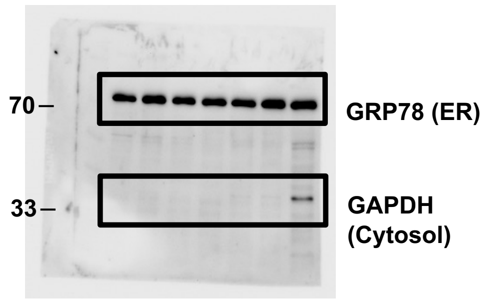

Calreticulin (ER)

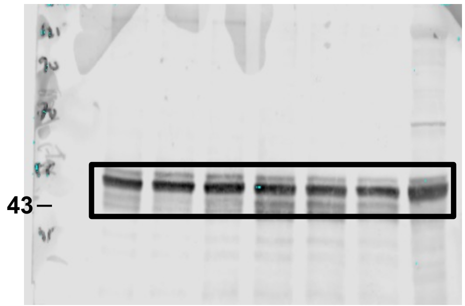

Lamp1 (lysosomes)

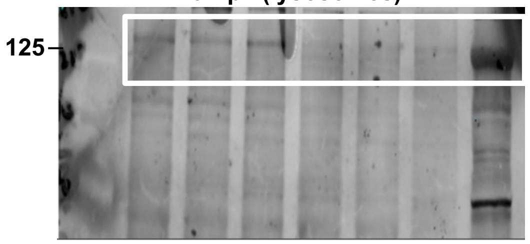

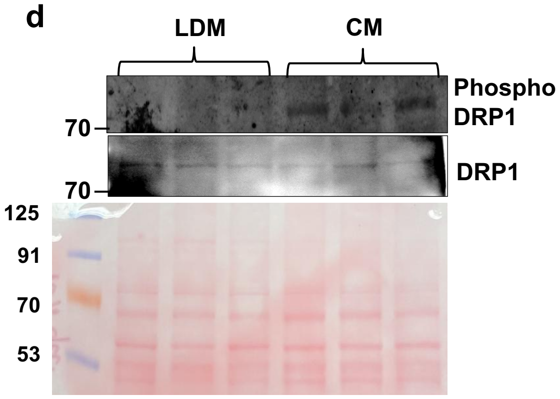

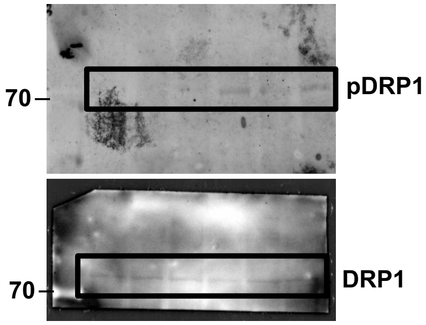

Uncropped blot images of supplementary Figure 3

Cropped image kept in manuscript

Uncropped image

a

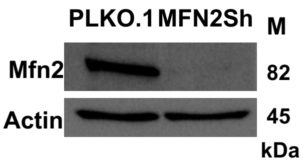

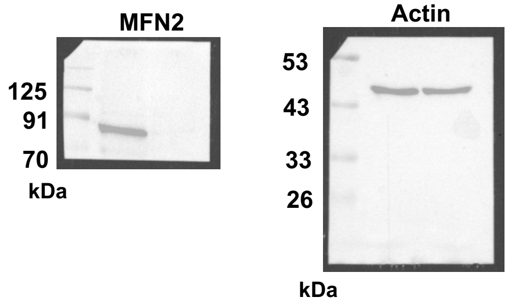

d

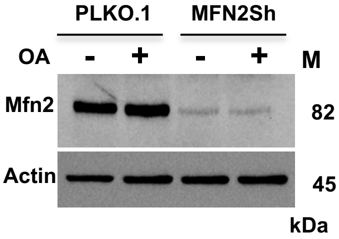

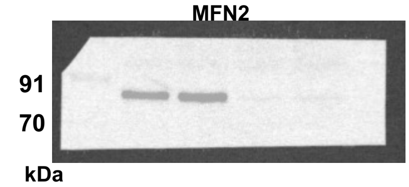

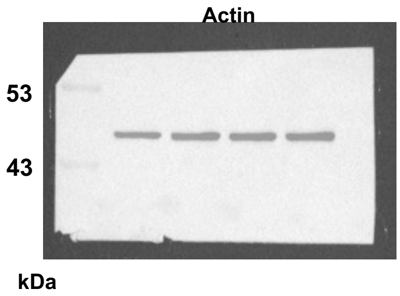

Uncropped blot images of supplementary Figure 3

Cropped image kept in manuscript

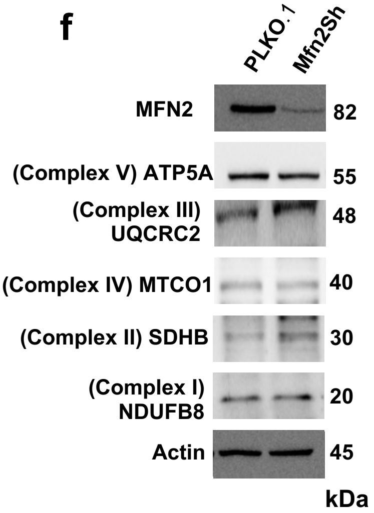

Uncropped image

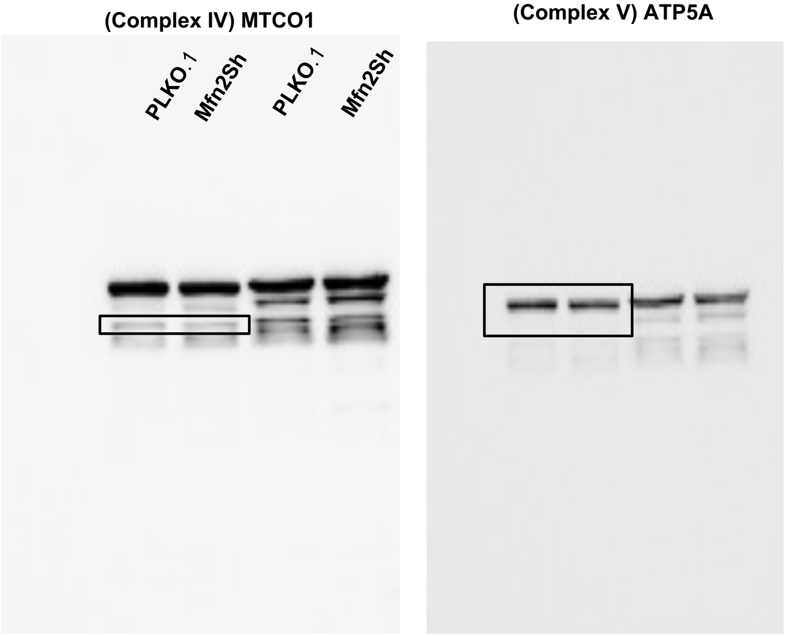

Uncropped image

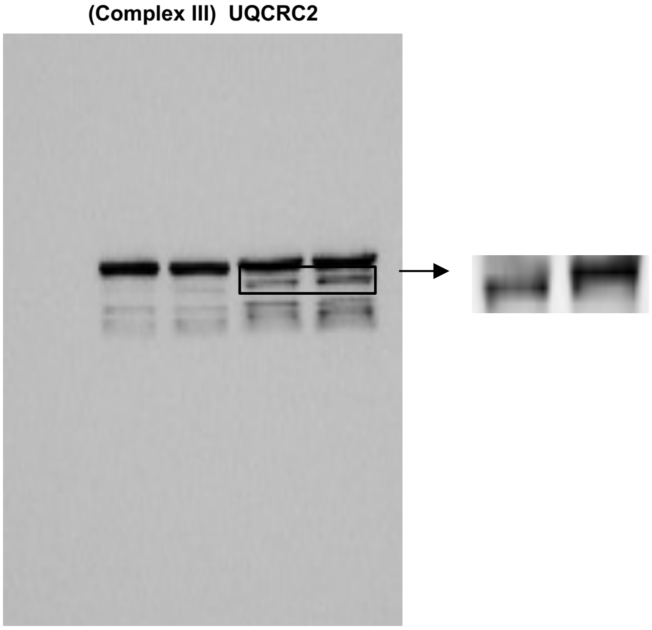

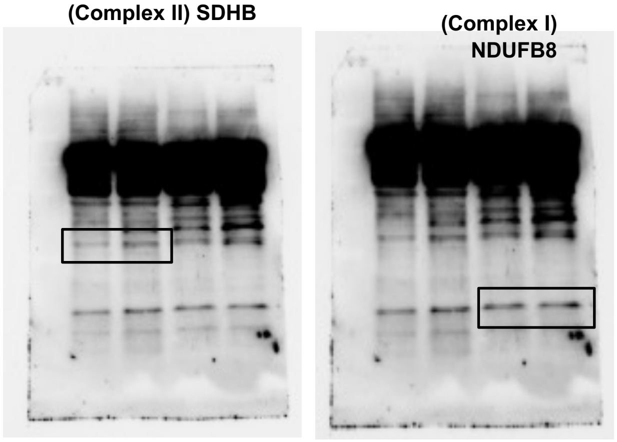

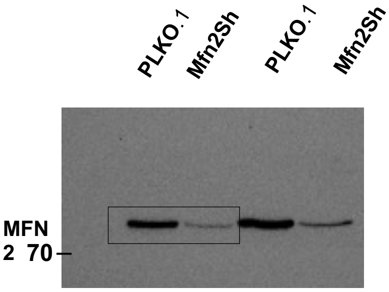

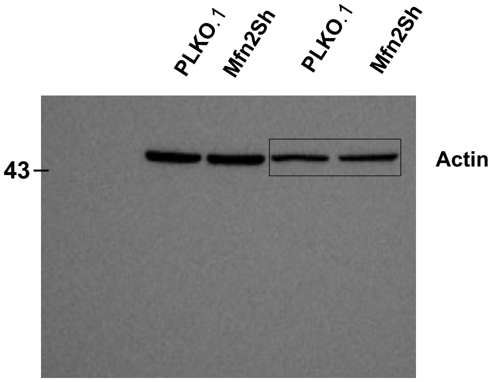

Uncropped blot images of supplementary Figure 3

Cropped image kept in manuscript

Uncropped image

h

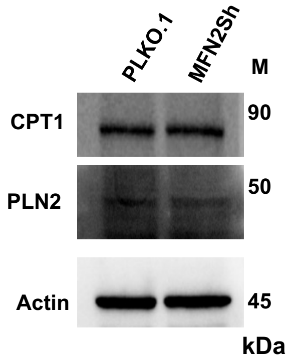

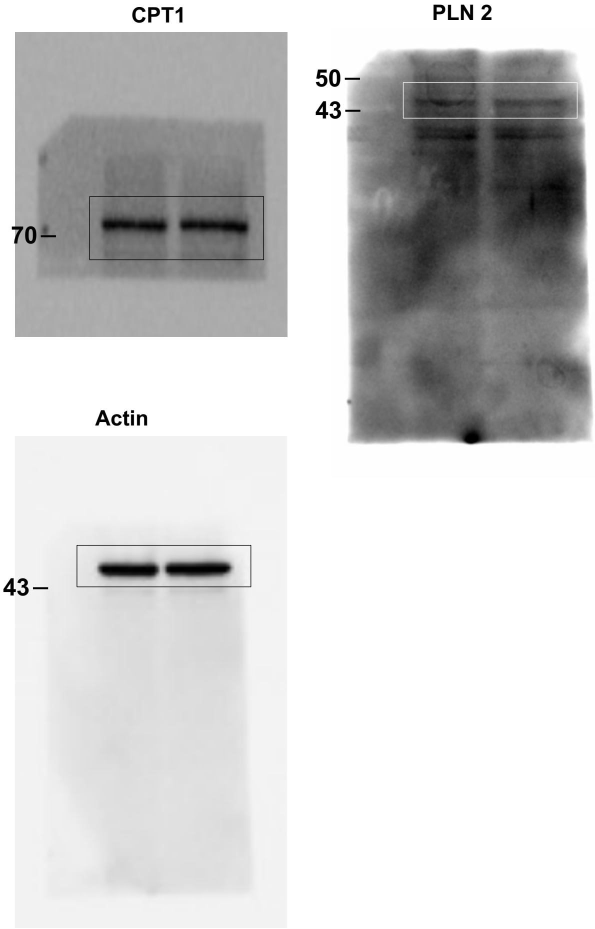

Supplement: Supplementary file 1 — Supplementary Information [file 41467_2023_36432_MOESM1_ESM.pdf]
