# Supplementary material for: Lipid-droplet associated mitochondria promote fatty-acid oxidation through a distinct bioenergetic pattern in male Wistar rats
Source: Nat Commun. 2023 Feb 11;14:766. doi: 10.1038/s41467-023-36432-0 (PMC9918515; doi:10.1038/s41467-023-36432-0)
Supplement: Supplementary file 3 — Reporting Summary [file 41467_2023_36432_MOESM3_ESM.pdf]

nature portfolio

Corresponding author(s): Naresh Babu V. Sepuri

Last updated by author(s): 22nd Jan 23

# Reporting Summary

Nature Portfolio wishes to improve the reproducibility of the work that we publish. This form provides structure for consistency and transparency in reporting. For further information on Nature Portfolio policies, see our Editorial Policies and the Editorial Policy Checklist.

## Statistics

For all statistical analyses, confirm that the following items are present in the figure legend, table legend, main text, or Methods section.

| n/a | Confirmed | |
|---|---|---|
| ☐ | ☒ | The exact sample size (*n*) for each experimental group/condition, given as a discrete number and unit of measurement |
| ☐ | ☒ | A statement on whether measurements were taken from distinct samples or whether the same sample was measured repeatedly |
| ☐ | ☒ | The statistical test(s) used AND whether they are one- or two-sided<br>*Only common tests should be described solely by name; describe more complex techniques in the Methods section.* |
| ☒ | ☐ | A description of all covariates tested |
| ☒ | ☐ | A description of any assumptions or corrections, such as tests of normality and adjustment for multiple comparisons |
| ☐ | ☒ | A full description of the statistical parameters including central tendency (e.g. means) or other basic estimates (e.g. regression coefficient) AND variation (e.g. standard deviation) or associated estimates of uncertainty (e.g. confidence intervals) |
| ☒ | ☐ | For null hypothesis testing, the test statistic (e.g. *F*, *t*, *r*) with confidence intervals, effect sizes, degrees of freedom and *P* value noted<br>*Give P values as exact values whenever suitable.* |
| ☒ | ☐ | For Bayesian analysis, information on the choice of priors and Markov chain Monte Carlo settings |
| ☒ | ☐ | For hierarchical and complex designs, identification of the appropriate level for tests and full reporting of outcomes |
| ☒ | ☐ | Estimates of effect sizes (e.g. Cohen's *d*, Pearson's *r*), indicating how they were calculated |

*Our web collection on statistics for biologists contains articles on many of the points above.*

## Software and code

Policy information about availability of computer code

| | |
|---|---|
| Data collection | Image Labs, (BioRad) for western blotting, Fluorescence Imaging was performed using a laser scanning confocal microscope (Model: NLO 710, CARL ZEISS). Image acquisition done by Zen black of Zeiss, Imaging performed on the JEOLJEM2100 TEM and Gatan digital micrograph software was used for image acquisition. SoftMax Pro is used in multiplate reader. |
| Data analysis | Image J version 1.5, GraphPad Prism 9.4.1, Fiji version 1.5(GitHub) |

For manuscripts utilizing custom algorithms or software that are central to the research but not yet described in published literature, software must be made available to editors and reviewers. We strongly encourage code deposition in a community repository (e.g. GitHub). See the Nature Portfolio guidelines for submitting code & software for further information.

## Data

Policy information about availability of data

All manuscripts must include a data availability statement. This statement should provide the following information, where applicable:

- Accession codes, unique identifiers, or web links for publicly available datasets
- A description of any restrictions on data availability
- For clinical datasets or third party data, please ensure that the statement adheres to our policy

The data supporting the findings of this work are available within the paper and the Supplementary Information files. Source data are provided as a source data file

## Human research participants

Policy information about studies involving human research participants and Sex and Gender in Research.

| | |
|---|---|
| Reporting on sex and gender | N/A |
| Population characteristics | N/A |
| Recruitment | N/A |
| Ethics oversight | N/A |

Note that full information on the approval of the study protocol must also be provided in the manuscript.

# Field-specific reporting

Please select the one below that is the best fit for your research. If you are not sure, read the appropriate sections before making your selection.

- [x] Life sciences
- [ ] Behavioural & social sciences
- [ ] Ecological, evolutionary & environmental sciences

For a reference copy of the document with all sections, see nature.com/documents/nr-reporting-summary-flat.pdf

# Life sciences study design

All studies must disclose on these points even when the disclosure is negative.

| | |
|---|---|
| Sample size | Sample sizes have been included in the Figure legends. Sample sizes were determined based on published literature and our preliminary studies. We ensured that the sample sizes were sufficient for statistical analyses without sacrificing too many animals. Experiments included at least three independent biological replicates. |
| Data exclusions | no data were excluded from the analysis |
| Replication | Information on the replicates have been included in the Figure legends |
| Randomization | animal groups used in the present study were divided into standard diet and high fat diet group selected randomly |
| Blinding | The investigators were blinded to group allocation during data collection and analysis |

# Reporting for specific materials, systems and methods

We require information from authors about some types of materials, experimental systems and methods used in many studies. Here, indicate whether each material, system or method listed is relevant to your study. If you are not sure if a list item applies to your research, read the appropriate section before selecting a response.

## Materials & experimental systems

| n/a | Involved in the study | |
|---|---|---|
| [ ] | [x] | Antibodies |
| [ ] | [x] | Eukaryotic cell lines |
| [x] | [ ] | Palaeontology and archaeology |
| [ ] | [x] | Animals and other organisms |
| [x] | [ ] | Clinical data |
| [x] | [ ] | Dual use research of concern |

## Methods

| n/a | Involved in the study | |
|---|---|---|
| [x] | [ ] | ChIP-seq |
| [x] | [ ] | Flow cytometry |
| [x] | [ ] | MRI-based neuroimaging |

## Antibodies

| | |
|---|---|
| Antibodies used | All antibodies used in this study are commercially available. Their catalogue numbers and their respective dilutions that were used have been mentioned in the manuscript.<br>Mitofusin2 (D2D10) Rabbit mAb Catalogue No. 9482 (Cell signaling Technologies). Dilution 1:1000<br>ACC (C83B10) Rabbit mAb Catalogue No. 3676 (Cell signaling Technologies). Dilution 1:1000<br>Phosphor-ACC (Ser79) (D7D11) Rabbit mAb Catalogue No. 11818 (Cell signaling Technologies). Dilution 1:1000;<br>Anti-CPT1A (8F6AE9) mouse mAb Catalogue No. ab128568 (Abcam). Dilution 1:2000 |

| | |
|---|---|
| | Total OXPHOS rodent WB antibody cocktail, Catalogue No. ab110413 (Abcam). Dilution 1:2000<br>Anti-ADFP (Perilipin2) (EPR3713) Rabbit mAb Catalogue No. ab108323 (Abcam). Dilution 1:2000<br>Anti-β-actin Catalogue No. A3854 (Sigma Aldrich). Dilution 1:10000<br>Drp1 rabbit Ab, Catalogue No. 8570 (Cell signaling Technologies). Dilution 1:1000<br>Phosphor-Drp1 (Ser616) Rabbit Ab, Catalogue No. 3455 (Cell signaling Technologies). Dilution 1:1000.<br>Calreticulin, Rabbit polyclonal Ab, Catalogue No. ab2907 (Abcam). Dilution 1:2000.<br>Lamp1, Rabbit polyclonal Ab, Catalogue No. ab62562 (Abcam). Dilution 1:2000.<br>GRP78, Rabbit polyclonal Ab, Catalogue No. ab21685 (Abcam). Dilution 1:2000.<br>GAPDH, mouse monoclonal Ab, Catalogue No. ab8245 (Abcam). Dilution 1:2000.<br>UCP2, rabbit monoclonal Ab, Catalogue No. 89326 (Cell signaling Technologies). Dilution 1:1000.<br>GDH, Rabbit Ab, Catalogue No. NB600-853 (Novus Biologicals). Dilution 1:2000.<br>Secondary Antibody: Peroxidase AffiniPure Goat Anti-Rabbit IgG (H+L), Cat No. 111-035-144 Dilution 1:10000, and Peroxidase AffiniPure Goat Anti-Mouse IgG (H+L), Cat No. 115-035-146 Dilution 1:10000 (Jackson Immuno Research Laboratories). |
| Validation | Validation statements has been mentioned by the respective manufacturers for the species used (rattus norvegicus) and application (western blotting). |

## Eukaryotic cell lines

Policy information about cell lines and Sex and Gender in Research

| | |
|---|---|
| Cell line source(s) | HEK293T cell line was purchased from the American Type Culture Collection (ATCC), USA. HepG2 cell line was purchased from the cell line repository of the National Centre for Cell Science (NCCS), India. |
| Authentication | Purchased from authenticated cell lines repository sources |
| Mycoplasma contamination | cell lines tested negative for mycoplasma contamination |
| Commonly misidentified lines (See ICLAC register) | No commonly misidentified cell lines were used in the study |

## Animals and other research organisms

Policy information about studies involving animals; ARRIVE guidelines recommended for reporting animal research, and Sex and Gender in Research

| | |
|---|---|
| Laboratory animals | Three-month-old male Wistar rats were used for all control experiments. Six-week-old male Wistar rats were fed with standard diet and high fat diet. |
| Wild animals | Study did not involve wild animals |
| Reporting on sex | Male |
| Field-collected samples | Study did not involve samples collected from field |
| Ethics oversight | All experiments were performed according to the Indian Institutional Ethical Committee board guidelines and approved by Institutional Animal Ethics Committee of University of Hyderabad, Hyderabad, India (UH/IAEC/NBVS/2021-22/12). |

Note that full information on the approval of the study protocol must also be provided in the manuscript.
